# β_3_ Relaxant Effect in Human Bladder Involves Cystathionine γ-Lyase-Derived Urothelial Hydrogen Sulfide

**DOI:** 10.3390/antiox11081480

**Published:** 2022-07-28

**Authors:** Emma Mitidieri, Annalisa Pecoraro, Erika Esposito, Vincenzo Brancaleone, Carlotta Turnaturi, Luigi Napolitano, Vincenzo Mirone, Ferdinando Fusco, Giuseppe Cirino, Raffaella Sorrentino, Giulia Russo, Annapina Russo, Roberta d’Emmanuele di Villa Bianca

**Affiliations:** 1 Department of Pharmacy, School of Medicine and Surgery, University of Naples Federico II, 80131 Naples, Italy; emma.mitidieri@unina.it (E.M.); annalisa.pecoraro@unina.it (A.P.); erika.esposito4@studenti.unina.it (E.E.); carlotta.turnaturi@unina.it (C.T.); cirino@unina.it (G.C.); giulia.russo@unina.it (G.R.); annapina.russo@unina.it (A.R.); demmanue@unina.it (R.d.d.V.B.); 2 Interdepartmental Centre for Sexual Medicine, University of Naples Federico II, 80131 Naples, Italy; vincenzo.mirone@unina.it; 3 Department of Science, University of Basilicata, 85100 Potenza, Italy; vincenzo.brancaleone@unibas.it; 4 Department of Neurosciences, Sciences of Reproduction, and Odontostomatology, University of Naples Federico II, 80131 Naples, Italy; luigi.napolitano@unina.it; 5 Department of Woman, Child and General and Specialized Surgery, Urology Unit, University of Campania Luigi Vanvitelli, 80131 Naples, Italy; ferdinando.fusco@unicampania.it; 6 Department of Molecular Medicine and Medical Biotechnology, School of Medicine, University of Naples Federico II, 80131 Naples, Italy

**Keywords:** hydrogen sulfide, urothelium, urothelium-derived relaxing factor, bladder, β_3_ adrenoceptor

## Abstract

It is now well established that the urothelium does not act as a passive barrier but contributes to bladder homeostasis by releasing several signaling molecules in response to physiological and chemical stimuli. Here, we investigated the potential contribution of the hydrogen sulfide (H_2_S) pathway in regulating human urothelium function in β_3_ adrenoceptor-mediated relaxation. The relaxant effect of BRL 37344 (0.1–300 µM), a selective β_3_ adrenoceptor agonist, was evaluated in isolated human bladder strips in the presence or absence of the urothelium. The relaxant effect of BRL 37344 was significantly reduced by urothelium removal. The inhibition of cystathionine-γ-lyase (CSE), but not cystathionine-β-synthase (CBS), significantly reduced the BRL 37344 relaxing effect to the same extent as that given by urothelium removal, suggesting a role for CSE-derived H_2_S. β_3_ adrenoceptor stimulation in the human urothelium or in T24 urothelial cells markedly increased H_2_S and cAMP levels that were reverted by a blockade of CSE and β_3_ adrenoceptor antagonism. These findings demonstrate a key role for urothelium CSE-derived H_2_S in the β_3_ effect on the human bladder through the modulation of cAMP levels. Therefore, the study establishes the relevance of urothelial β_3_ adrenoceptors in the regulation of bladder tone, supporting the use of β_3_ agonists in patients affected by an overactive bladder.

## 1. Introduction

The urothelium is a stratified epithelium that has been thought, for a long time, to act as a barrier, protecting the bladder’s smooth muscle layer against irritating urine constituents [1]. In more recent years it has been demonstrated that it actively participates in bladder homeostasis. Indeed, several pieces of evidence show that the contractions elicited by tachykinins, muscarinic agonists, or potassium chloride are strongly enhanced in the detrusor muscle after urothelium removal in several species, i.e., rat, guinea pig, pig, dog, and human [2,3,4,5,6,7,8,9,10,11,12]. It is now well established that the urothelium contributes to bladder tone by dynamically modulating both the contracting and relaxant responses, essentially behaving like the vascular endothelium. The urothelium releases several mediators, including ATP, acetylcholine, prostaglandins, nitric oxide, and nerve growth factor, affecting bladder tone and function, smooth muscle cell growth, and the afferent nerve [13]. The contribution of the urothelium in regulating bladder tone has also been ascribed to the release of a diffusible factor, defined as a urothelium-derived relaxing factor (UDRF); the molecular identity of UDRF currently remains elusive. A large body of evidence has ruled out several mediators as possible UDRFs, including nitric oxide, cyclooxygenase products, catecholamines, adenosine and GABA or openers of purinergic P2Y receptors, TEA-sensitive K+ channels, and small-conductance Ca^2+^-activated K^+^ channels. [5,6,10,14,15]. More recently, hydrogen sulfide (H_2_S) has been proposed as a feasible UDRF candidate [12,16]. H_2_S is endogenously produced in the urinary bladder of several species, such as trout, mouse, rat, pig, and human, and the fact that it is phylogenetically conserved implies that it has a key role in the control of bladder function [17,18,19,20,21,22]. Cystathionine-β-synthase (CBS), cystathionine-γ-lyase (CSE), and 3-mercaptopyruvate sulfurtransferase (3-MST), the three major enzymes responsible for the endogenous production of H_2_S, are expressed in the human bladder [19,21]. Among these, CBS and CSE are pyridoxal-phosphate (PLP)-dependent enzymes in the transsulfuration pathway and are involved in homocysteine metabolism [23]. In more detail, CBS catalyzes the conversion of homocysteine in cystathionine, whilst CSE synthesizes L-cysteine from cystathionine. The third H_2_S-generating enzyme is 3-MST, which participates in cysteine metabolism in a PLP-independent manner. It mainly resides within mitochondria and acts in conjunction with cysteine aminotransferase (CAT) to produce H_2_S [24]. The human bladder expresses CBS and CSE and efficiently converts L-cysteine into H_2_S [21]. Moreover, sodium hydrogen sulfide and L-cysteine (exogenous and endogenous sources of H_2_S, respectively) relax in a concentration-dependent manner human bladder strips precontracted with carbachol, indicating a relevant role for H_2_S in bladder homeostasis [21]. This hypothesis is further sustained by the finding that pharmacological modulation with CBS and/or CSE inhibitors increases the carbachol response in isolated bladder strips with intact urothelium [12].

Although β_3_ agonists are currently used for the treatment of overactive bladder, the site and mechanism of action of such agonists are still open questions [2]. It is a well-consolidated concept that the effect of the β_3_ adrenoceptor (AR) occurs mainly through a direct relaxant effect of the detrusor muscle due to the activation of cAMP and Ca^2+^-activated K^+^ channels [25,26]. However, studies have suggested that cAMP signaling is not the only mechanism of β_3_ AR activation [27,28,29]. Indeed, more recently, UDRF has been proposed to be involved in β_3_ AR-induced relaxation in the human bladder [30].

Since it is known that (i) β_3_ AR is expressed in the human detrusor and urothelium [31,32], and (ii) β_3_ AR stimulation relaxes the human corpus cavernosum through H_2_S release [33], here, we have investigated the contribution of the urothelium and the possible involvement of the H_2_S pathway in β_3_-induced relaxation in the human bladder. 

## 2. Materials and Methods

### 2.1. Human Tissue

Bladder samples were obtained from 15 male patients (aged 61–73 years ) who underwent open prostatectomy or trans-urethral bladder resection. Tissue harvesting and experimental procedures, performed following the Declaration of Helsinki (2013) of the World Medical Association, were approved by the Ethical Committee of the institution (School of Medicine and Surgery, University of Naples Federico II, via Pansini, 5; 80131, Naples, Italy). Informed consent was obtained from all subjects involved in the study.

### 2.2. Human Bladder Strips

Human bladder samples were collected and longitudinal strips were isolated [12]. Strips were placed in organ chambers (3 mL) filled with oxygenated (95% oxygen and 5% carbon dioxide) Krebs buffer (sodium chloride, 115.3 mM; potassium chloride, 4.9 mM; calcium chloride, 1.46 mM; magnesium sulfate, 1.2 mM; potassium dihydrogen phosphate, 1.2 mM; sodium bicarbonate, 25.0 mM; and glucose, 11.1 mM; Carlo Erba, Milan, Italy) at 37 °C. Strips were connected to isometric transducers (FORT25, World Precision Instruments, 2Biological Instruments, Besozzo, VA, Italy) associated with Power Lab 8/35 (2Biological Instruments, Besozzo VA, Italy). Tissues were stretched to a resting tension of 0.5 g and, after equilibration (60 min), were standardized via repeated carbachol (1 µM; Sigma, Italy) contractions [12]. A cumulative concentration–response curve for BRL 37344 (0.1–300 µM, Tocris, UK), a β_3_ AR selective agonist, was performed on strips pre-contracted with carbachol, in the presence or absence of urothelium. In another set of experiments, the strips were pretreated with DL-propargylglycine (PAG; 60 µL to reach 10 mM; Sigma, Milan, Italy) or aminooxy acetic acid (AOAA; 3 µL to reach 1 mM; Sigma, Milan, Italy), inhibitors for CSE and CBS, respectively, before the BRL 37344 challenge in the presence or absence of urothelium. Data were calculated as a percentage of relaxation of the carbachol stable tone and expressed as the mean ± SEM (*n* = 5). The results were analyzed using analysis of variance (ANOVA) followed by the Bonferroni post hoc test. *p* < 0.05 was considered significant.

### 2.3. Human Urothelial T24 Cells

T24 cell lines were cultured under a humidified atmosphere of 5% CO_2_ at 37 °C in Dulbecco’s Modified Eagle Medium (DMEM) enriched with 10% heat-inactivated fetal bovine serum (FBS) (Invitrogen, Life Technologies, Milan, Italy), 1.5 mM L-glutamine, 100 units/mL penicillin, and 100 μg/mL streptomycin [12]. Treatments of cells were performed by replacing the culture medium with those containing increasing concentrations of BRL 37344 (0.1–100 µM), PAG (10 mM), SR59230A (10 µM), or AOAA (1 mM).

### 2.4. Western Blot

Samples of human urothelium or T24 cells were homogenized in modified RIPA buffer (Tris-HCl 50 mM pH 8.0, NaCl 150 mM, sodium deoxy-cholate 0.5%, sodium dodecyl sulfate 0.1%, EDTA 1 mM, Igepal 1%) (Roche Applied Science, Monza, Italy) and protease inhibitor cocktail (Sigma, Milan, Italy). Protein concentration was determined using the Bradford assay, using albumin (BSA, Sigma, Milan, Italy) as standard. Denatured proteins (50 µg) were separated on 10% sodium dodecyl sulfate-polyacrylamide gels and transferred to a polyvinylidene fluoride membrane [34]. The membranes were blocked for 1 h at room temperature in phosphate buffer solution (PBS) containing 0.1% *v*/*v* Tween 20 and 5% non-fat dried milk; thereafter, they were incubated overnight at 4 °C with mouse monoclonal antibody for β_3_ AR (1:1000; MyBioSource, Bergamo, Italy). The same membranes were stripped, cut to 50 kDa; then, the upper sections were incubated with mouse monoclonal antibody for CSE (1:1000; Abnova, Milan, Italy), and the bottom with rabbit polyclonal for CBS (1:1000; Santa Cruz Biotechnology, Inc., Dallas, TX, USA) overnight at 4 °C. Membranes were extensively washed in PBS containing 0.1% *v*/*v* Tween 20 (four times, once every five minutes) and then incubated with horseradish peroxidase-conjugated secondary antibody for 2 h at room temperature. Following incubation, membranes were washed and developed using Chemidoc (Bio-Rad, Milan, Italy). The target protein band intensity was normalized against the intensity of the housekeeping protein ß-actin (1:5000, Sigma-Aldrich, Milan, Italy).

### 2.5. H_2_S Determination

To assess the activity of CBS and CSE in the human urothelium and T24 cells, H_2_S determination was evaluated in basal conditions and after the addition of L-cysteine, as previously reported [35]. Samples of detrusor muscle were incubated with vehicle or BRL 37344 at concentrations of 0.1–10 µM for 5 min. In another set of experiments, samples of human urothelium were incubated with vehicle or BRL 37344 (0.1–10 µM) for 5 min. A concentration of 1 µM was chosen for the following experiments in urothelium homogenates. The samples were incubated for 20 min with AOAA (1 mM), a CBS inhibitor; PAG (10 mM), a CSE inhibitor; or SR59230A (10 µM; Sigma, Milan, Italy), a β_3_-selective antagonist, and then stimulated with BRL 37344 (1 µM). To better define the mechanism beyond β_3_ AR activation, the same experimental protocol was performed in T24 cells. Frozen-kept samples were used to measure H_2_S levels [36]. Briefly, samples were lysed in an appropriate buffer (potassium phosphate buffer 100 mM, pH 7.4, sodium orthovanadate 10 mM, and protease inhibitors) and protein concentration was determined using the Bradford assay (Bio-Rad Laboratories, Milan, Italy). The reaction was performed in sealed Eppendorf tubes and initiated by transferring tubes from ice to a water bath at 37 °C for 30 min. Next, trichloroacetic acid solution (10% wt/vol) was added to each sample, followed by zinc acetate (1% wt/vol). Subsequently, *N*,*N*-dimethyl-*p*-phenylendiamine sulfate (DPD; 20 mM) in HCl (7.2 M) and FeCl_3_ (30 mM) in HCl (1.2 M) were added, and optical absorbance of the solution was measured after 20 min at a wavelength of 668 nm. All samples were assayed in duplicate, and H_2_S concentrations were calculated against a calibration curve of NaHS (3–250 µM). Data were calculated as nanomoles/mg/min of protein and expressed as the mean ± SEM (n = 5 for human detrusor muscle; n = 6 for human urothelium; n = 5 for T24 cells). The results were analyzed using analysis of variance (ANOVA) followed by the Bonferroni post hoc test. *p* < 0.05 was considered significant.

### 2.6. Determination of cGMP and cAMP in T24 Cells

Cyclic guanosine monophosphate (cGMP) and cyclic adenosine monophosphate (cAMP) content were measured in samples of T24 cells incubated with vehicle or BRL 37344 at concentrations of 0.1–100 µM for 5 min. cAMP content was measured in T24 cells incubated for 20 min with AOAA (1 mM), a CBS inhibitor; PAG (10 mM), a CSE inhibitor; or SR59230A (10 µM; Sigma, Milan, Italy), a selective β_3_ AR antagonist before stimulation with BRL 37344 (10 µM). cGMP and cAMP contents were measured as described in the manufacturer’s protocol for the cGMP and cAMP EIA Kit (Cayman, Vinci-Biochem, Vinci, Italy) [37,38]. All samples were assayed in duplicate and cyclic nucleotide concentrations were calculated against a calibration curve of standard cGMP or cAMP. Data were calculated as pmol/mL. Results were expressed as mean ± SEM (n = 5) and analyzed using analysis of variance (ANOVA) followed by the Bonferroni post hoc test. *p* < 0.05 was considered significant.

## 3. Results

### 3.1. β3 AR, CBS, and CSE Are Expressed in the Human Urothelium and T24 Cells

**Figure 1:**
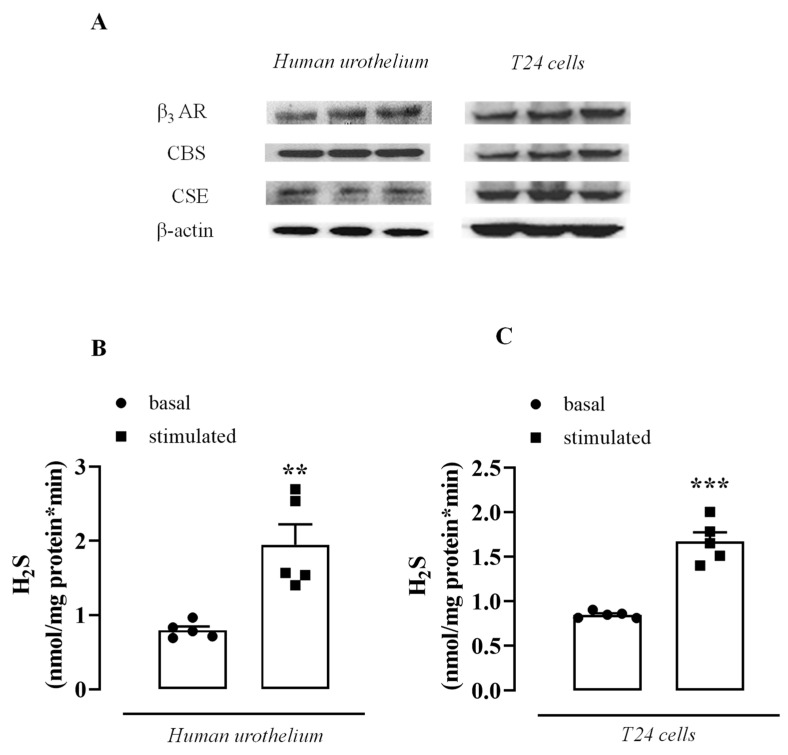
β_3_ AR and H_2_S signaling in the human urothelium and T24 cells. (**A**) Representative Western blot for β_3_ AR, CBS, and CSE in the human urothelium and T24 cells. β-actin his considered the housekeeping protein. (**B**) Generation of basal or stimulated (L-cysteine addition) H_2_S in human urothelium (** *p* < 0.01). (**C**) Generation of basal or stimulated (L-cysteine addition) H_2_S in T24 cells (*** *p* < 0.001).

Human urothelium and T24 cells express β_3_ AR (Figure 1A) as well as the key enzymes involved in endogenous H_2_S biosynthesis, CBS, and CSE (Figure 1A). Human urothelium and T24 cells can functionally convert L-cysteine into H_2_S. The basal rate of the H_2_S generated was increased 2.5 and 2 times in the human urothelium and T24 cells, respectively, following the addition of L-cysteine, i.e., in the stimulated condition (Figure 1B,C, ** *p* < 0.01; *** *p* < 0.001). 

### 3.2. BRL 37344-Induced Relaxation Involves H_2_S Production in Human Bladder Strips

**Figure 2:**
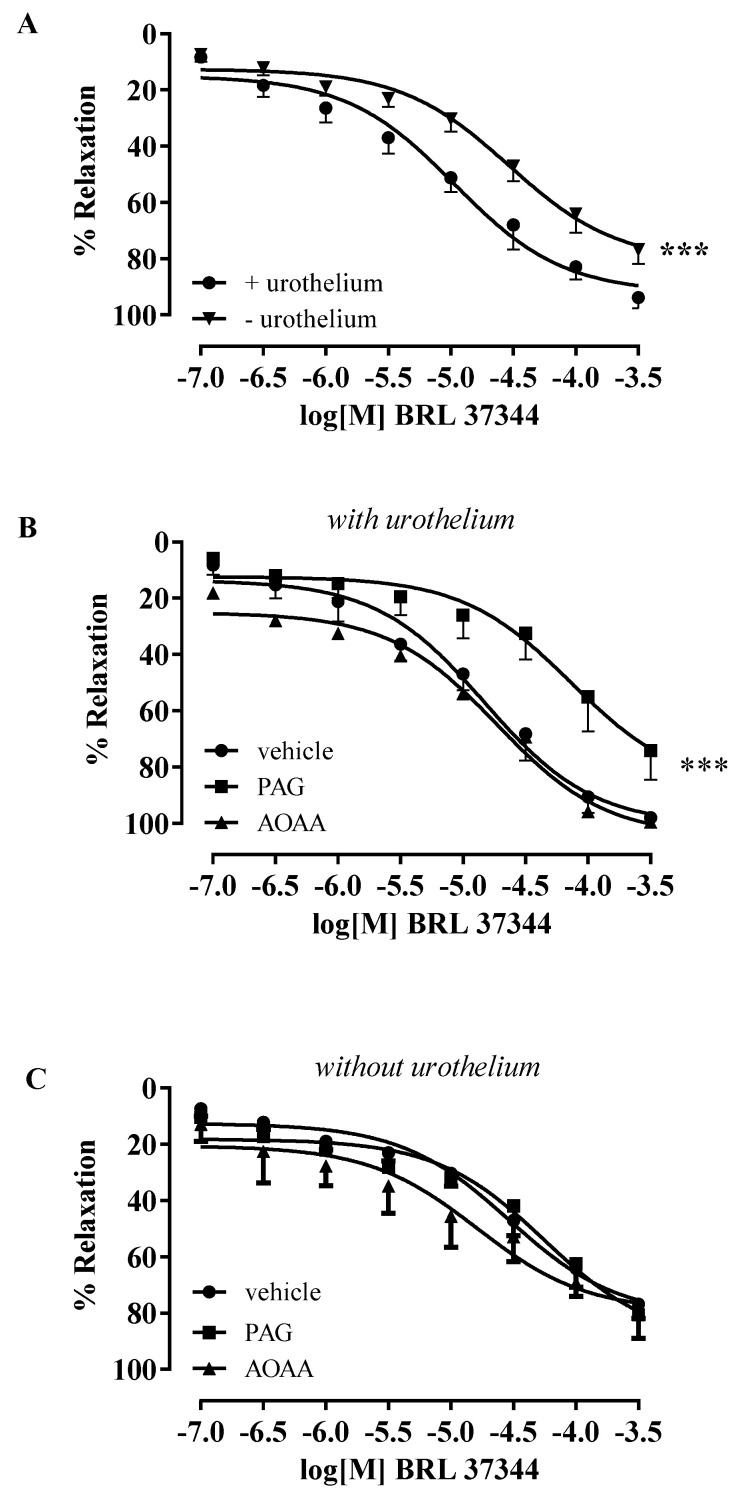
Effect of BRL 37344 on isolated human bladder strips. (**A**) BRL 37344 relaxed human bladder strips in a concentration-dependent manner. The relaxant effect of BRL 37344 was significantly higher in strips with intact urothelium compared to denuded strips (*** *p* < 0.001). (**B**) The relaxant effect of BRL 37344 was significantly reduced by PAG in strips with intact urothelium (*** *p* < 0.001). AOAA did not modify the relaxant effect of BRL 37344. (**C**) The relaxant effect of BRL 37344 was not affected by AOAA or PAG in denuded strips.

On the stable tone of carbachol, a cumulative concentration–response curve for BRL 37344 (0.1–300 µM) was performed on strips with intact urothelium or in urothelium-denuded strips. BRL 37344 elicited significantly higher concentration-dependent relaxation in strips with intact urothelium (*** *p* < 0.001, Figure 2A). To evaluate the involvement of H_2_S, the strips were incubated with AOAA (CBS inhibitor) or PAG (CSE inhibitor). The BRL 37344 relaxant effect in isolated strips with intact urothelium was significantly reduced following CSE inhibition with PAG, while no changes were observed after AOAA treatment (*** *p* < 0.001, Figure 2B). Conversely, in denuded strips, neither the CBS nor the CSE inhibitors modified the relaxant effect of BRL 37344 (Figure 2C).

### 3.3. BRL 37344 Promotes H_2_S Production in the Human Urothelium and T24 Cells

**Figure 3:**
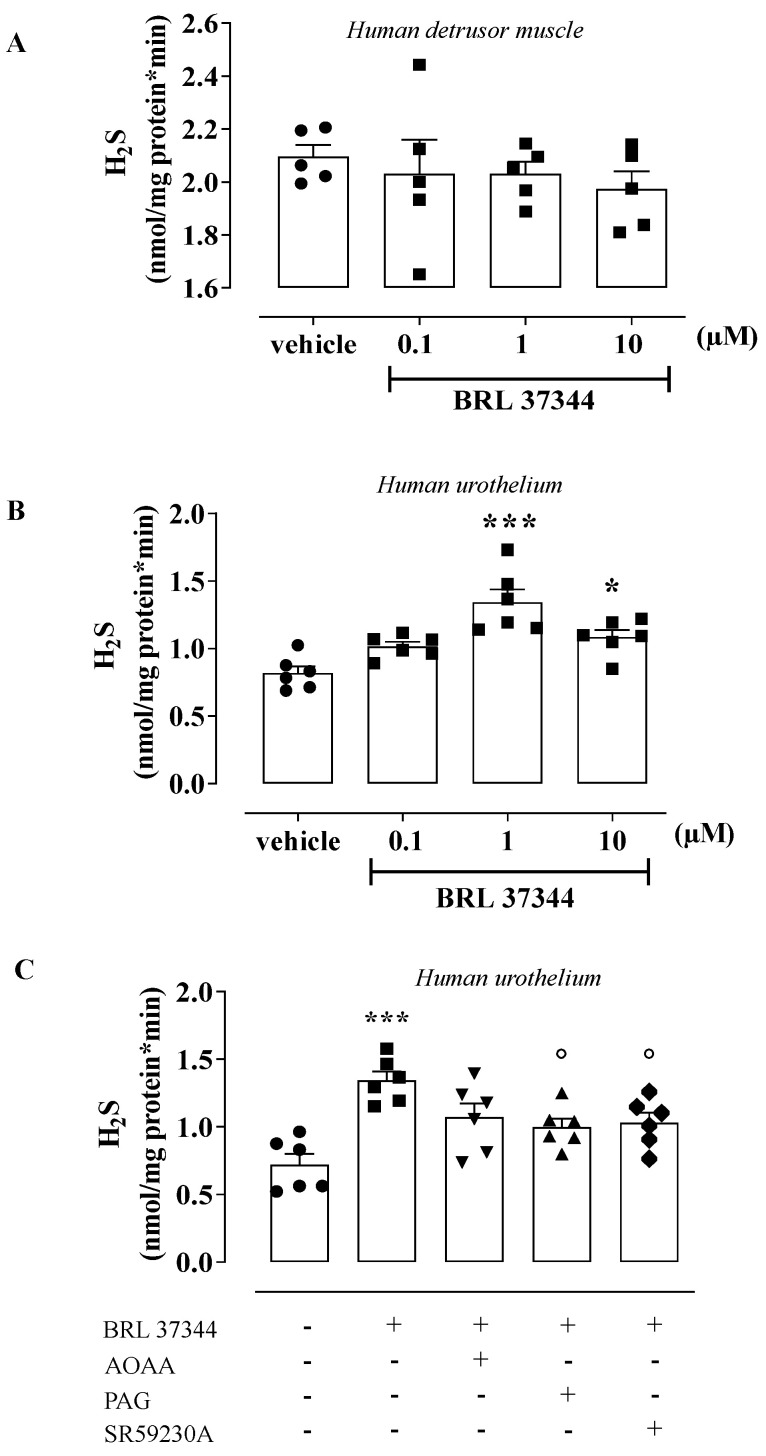
Effect of BRL 37344 on H_2_S production in the human bladder. (**A**) BRL 37344 (0.1–10 µM) did not modify the H_2_S rate of production compared to vehicle in human detrusor muscle. (**B**) BRL 37344 at 1 and 10 µM increased the H_2_S rate of production 1.7 and 1.4 times, respectively, compared to vehicle in the human urothelium (*** *p* < 0.001 and * *p* < 0.05). (**C**) PAG (10 mM) and SR59230A (10 µM) reduced BRL 37344 (1 µM)-induced H_2_S production 0.7-fold in the human urothelium (*** *p* < 0.001 vs. vehicle; ° *p* < 0.05 vs. BRL 37344); AOAA (1 mM) did not affect the BRL 37344-induced effect.

**Figure 4:**
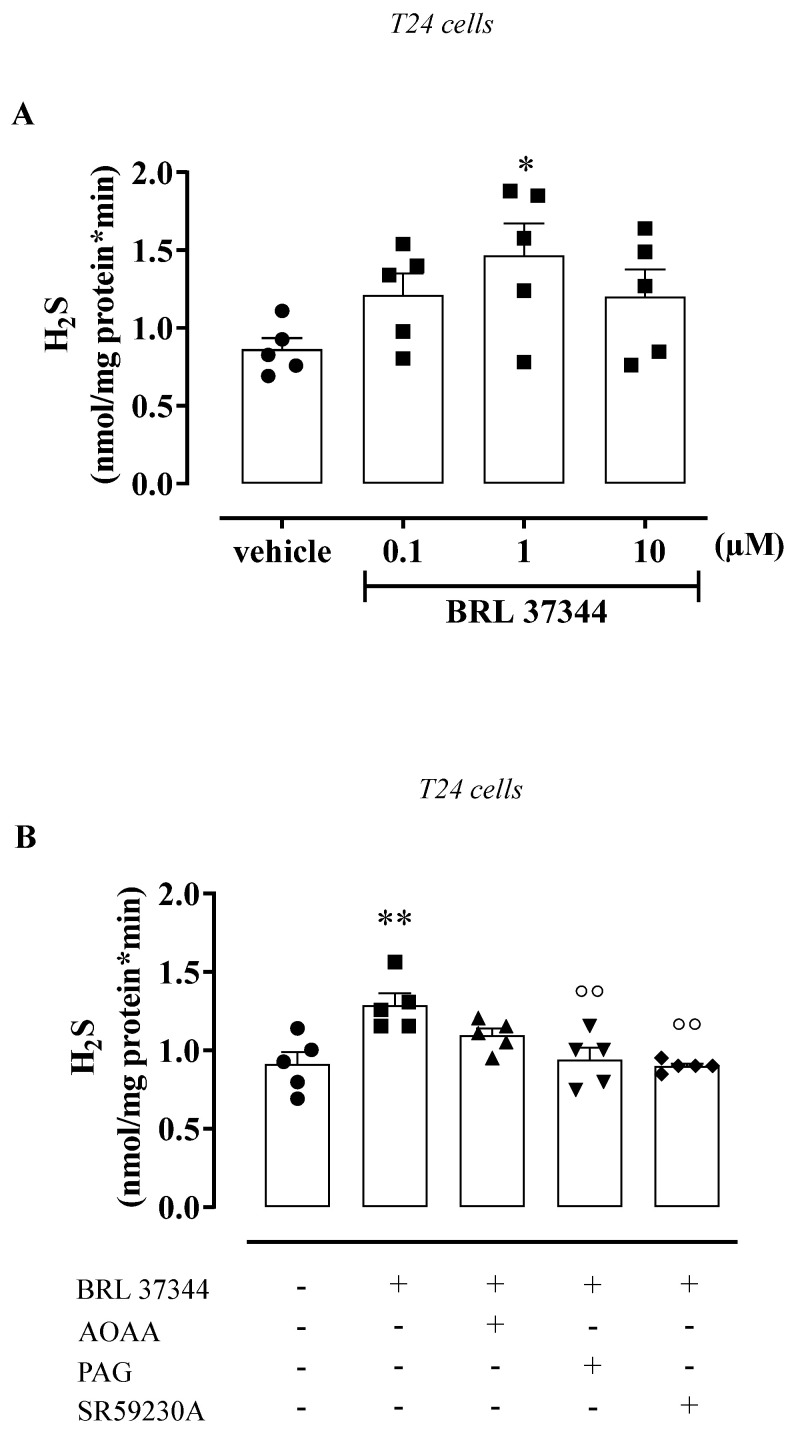
Effect of BRL 37344 on H_2_S production in T24 cells. (**A**) BRL 37344 (1 µM) increased the H_2_S rate of production 1.7 times compared to vehicle in T24 cells (* *p* < 0.05). (**B**) PAG (10 mM) and SR59230A (10 µM) reduced BRL 37344 (1 µM)-induced H_2_S production 0.7-fold (** *p* < 0.01 vs. vehicle; °° *p* < 0.01 vs. BRL 37344); AOAA did not affect the BRL 37344-induced effect.

To assess the H_2_S contribution in β_3_ AR stimulation, samples of the human detrusor muscle and urothelium were incubated with BRL 37344 (0.1–10 µM) or a vehicle for 5 min. BRL 37344 (0.1–10 µM) did not affect the H_2_S rate of production in human detrusor muscle (Figure 3A). Conversely, BRL 37344 caused a 1.7-fold increase in the H_2_S rate of production in the human urothelium, reaching the maximum effect at 1 µM (*** *p* < 0.001, Figure 3B). Based on this evidence, a concentration of 1 µM was chosen as the best concentration to use. The increase in the H_2_S rate of production induced by BRL 37344 (1 µM) was reduced 0.7 times following pretreatment with PAG, CSE inhibitor, or a β_3_ AR antagonist, namely SR59230A (° *p* < 0.05, Figure 3C); the AOAA pretreatment (CBS inhibitor) did not affect the increase in H_2_S production. To better define the involvement of H_2_S signaling in β_3_ AR activation in the urothelium, T24 cells were used. T24 cells stimulated with BRL 37344 (0.1–10 µM) showed the same profile as that observed in the human urothelium (Figure 4). Indeed, the highest peak of the H_2_S rate of production was achieved with BRL 37344 1 µM (* *p* < 0.05, Figure 4A), and this effect was reverted by PAG or SR59230A (°° *p* < 0.01, Figure 4B), but not by AOAA.

### 3.4. BRL 37344 Increases cAMP Levels in T24 Cells

**Figure 5:**
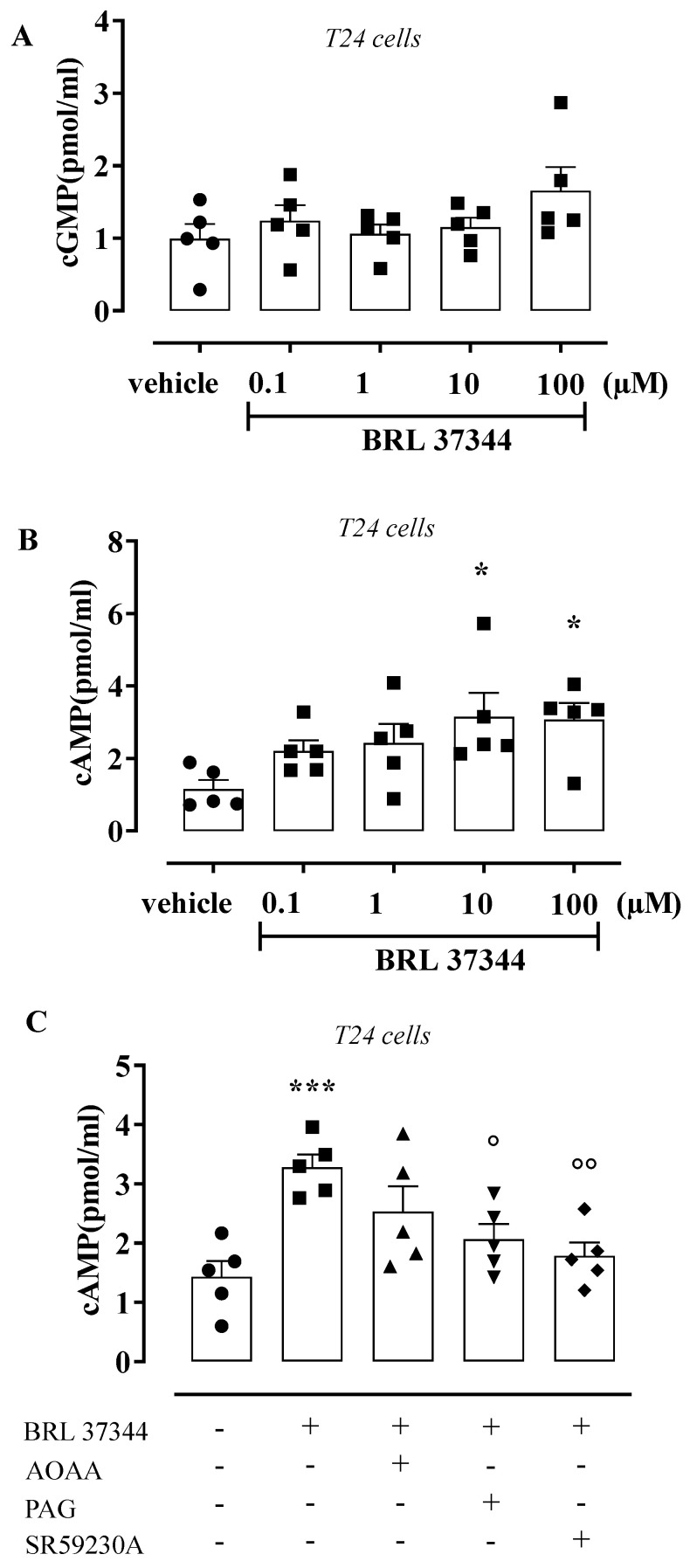
Effect of BRL 37344 on cGMP and cAMP content in T24 cells. (**A**) BRL 37344 (0.1–100 µM) did not modify cGMP content compared to vehicle in T24 cells. (**B**) BRL 37344 (0.1–100 µM) increased the cAMP content 2.7 times compared to vehicle in T24 cells (* *p* < 0.05). (**C**) PAG (10 mM) and SR59230A (10 µM), but not AOAA (1 mM), reduced the BRL 37344 (1 µM)-induced cAMP content (*** *p* < 0.001 vs. vehicle; ° *p* < 0.05 and °° *p* < 0.01 vs. BRL 37344) 0.63- and 0.54-fold.

To evaluate the downstream messenger coupled with β_3_ AR activation in the urothelium, the levels of cGMP and cAMP were measured in T24 cells incubated with BRL 37344 (0.1–100 µM). The challenge with BRL 37344 did not affect the cGMP production (Figure 5A). Contrariwise, cAMP levels were increased 2.7 times, reaching the maximum effect at 10 µM (* *p* < 0.05, Figure 5B). Under this experimental condition, 10 µM was chosen as the best concentration to use for pharmacological modulation. The increase in cAMP content induced by BRL 37344 (10 µM) resulted in a 0.63- and 0.54-fold reduction following pretreatment with PAG and SR59230A, respectively (° *p* < 0.05; °° *p* < 0.01, Figure 5C), but was not affected by AOAA.

## 4. Discussion

The urothelium is a highly specialized tissue that not only performs as a barrier to urine, but also stretches when the bladder fills and contracts when it empties. Its role as a simple “bystander” has recently been challenged, and the urothelium has emerged as a feasible additional cellular target [30]. The β_3_ subtype receptor predominates over β_1_ and β_2_ subtypes in the human bladder in both urothelium and detrusor muscle [39,40,41,42]. The development of β_3_ AR agonists for the treatment of overactive bladder syndrome has further stressed the key role of this receptor subtype in bladder physiopathology [43,44,45,46,47,48,49]. Despite their clinical success, controversies exist concerning their molecular mechanism(s) of action [30,50,51,52]. The current proposed mechanism is a direct relaxation of the detrusor muscle driven by the activation of β_3_ AR. The role of the urothelium in the β_3_ agonist effect has not been addressed. In this scenario, we have previously shown that the activation of muscarinic receptors within the urothelium promotes H_2_S generation [12]. This evidence leads us to hypothesize a possible link between the urothelium/β_3_ AR and H_2_S. 

Here, we have clarified that β_3_ AR is expressed in the human urothelium and T24 cells and confirmed the presence of the two main enzymes producing H_2_S, namely CBS and CSE. The biological assay performed using the human urothelium as an enzymatic source also demonstrates the presence of a basal H_2_S tone which, upon incubation with the substrate, i.e., L-cysteine, increases 2.5 times. These in vitro data indicated that in the human bladder, there exists a physiological H_2_S tone, and that the enzymes present within the tissue are active. Having acquired this biochemical information to aid our understanding of whether this production of H_2_S plays a physiological role, we performed a functional study, i.e., an organ bath. As expected, we found that β_3_ AR activation by the agonist BRL 37344 led to concentration-dependent relaxation of the human-isolated bladder strips. However, when we removed the urothelium, there was a significant reduction in the BRL 37344 relaxing effect. Thus, β_3_ AR activation involves both the detrusor and the urothelium. 

To define the possible role played by the H_2_S pathway in the β_3_ agonist effect on the urothelium, we performed a pharmacological modulation study by using PAG as a CSE inhibitor and AOAA as a CBS inhibitor. We demonstrated that in human strips, urothelium CSE-derived H_2_S plays a key role. Indeed, while AOAA was ineffective, PAG caused a marked reduction in the relaxing effect elicited by the β_3_ AR agonist. In more detail, the EC50 of BRL 37344 was 14.9 ± 1.35 µM, increasing to 73.2 ± 1.88 µM in the presence of PAG. The CSE effect was lost upon urothelium removal. Thus, the β_3_ agonist effect involves the modulation of CSE-derived H_2_S produced within the urothelium. The bioassay does not allow us to measure relaxation and H_2_S production simultaneously. For this reason, we incubated human detrusor and the urothelium in vitro with BRL 37344. As expected, BRL 37344 did not increase H_2_S production in the detrusor, but only in the urothelium. The pharmacological modulation performed using the same inhibitors demonstrated that the BRL 37344 challenge induced CSE-derived H_2_S in the urothelium. This effect is dependent upon the activation of β3 AR on the urothelium, since the β_3_ AR antagonist SR59230A significantly inhibited this effect. Thus, the activation of β_3_ AR on the urothelium leads to an increase in CSE-derived H_2_S, which contributes to the relaxant effect. These findings converge with other pieces of evidence present in the relevant literature reporting that β_3_ AR activation in other districts, such as mouse stomach and human corpus cavernosum, involves H_2_S signaling [33,53]. It is feasible to speculate that the contribution of CBS and/or CSE in β_3_ AR activation may depend upon the district/organ. Indeed, in gastric fundal tissue, both CBS and CSE are involved, while in the human corpus cavernosum, CSE predominates, as we found in the human bladder. Thus, in the genito-urinary tract in men, CSE appears to be the main enzyme coupled with β_3_ AR in H_2_S production.

The activation of β_3_ AR increases cAMP levels [54]. To evaluate the involvement of cAMP and/or cGMP, due to the difficulty in obtaining fresh human tissue to run these experiments, we took advantage of the similitude between human urothelium and T24 cells. Indeed, the pattern observed in the urothelium was also evident in T24 cells. Indeed, incubation with BRL 37344 generated an increase in H_2_S production that was reverted by CSE or a β_3_ AR blockade but not by CBS inhibition. As expected, the incubation of T24 cells with the β_3_ AR agonist caused a concentration-dependent increase in cAMP levels without affecting cGMP. As observed in the functional studies, the blockade of CSE or β_3_ AR, but not CBS, leads to a significant reduction in cAMP levels in T4 cells. Therefore, the evidence showing that H_2_S can negatively modulate phosphodiesterase activity [55] also corroborates the existence of the link between β_3_ AR, CSE, and cAMP, which we propose here. Several studies showed a positive correlation between H_2_S and cAMP levels in several types of cell, such as neutrophils [56], platelets [57], vascular smooth muscle [58], and epithelial cells [59]. Interestingly, a lower activity of cAMP/PKA signaling was found in CSE-knockout hepatocytes, supporting the role of H_2_S in cAMP modulation [60]. 

Thus, we believe that β_3_ AR plays a major role within the urothelium, generating CSE-dependent myorelaxation through the release of H_2_S, responsible for the increase in cAMP levels following the inhibition of phosphodiesterases [55].

## 5. Conclusions

In conclusion, we have demonstrated that the human urothelium is involved in the relaxant effect of β_3_ AR agonists. The urothelium’s effect on bladder smooth muscle is driven by CSE-derived H_2_S release, which leads to an increase in cAMP. These findings imply that in physiological conditions, the contribution of the sympathetic tone through β_3_ receptors to bladder homeostasis involves both the urothelium and the smooth muscle component. Our study defines a new molecular mechanism underlying the β_3_ agonist effect in the human bladder that, at the present stage, is thought to be driven only by the smooth muscle component. Here, we show evidence that the β_3_ mechanism of action involves urothelial β_3_ receptors, leading to the release of CSE-derived H_2_S and, in turn, to an increase in cAMP.

## Data Availability

Data is contained within the manuscript.
